# Supplementary material for: Evaluation of rapid diagnostic test kits for detection of Treponema pallidum antibody
Source: PLoS One. 2024 May 29;19(5):e0303477. doi: 10.1371/journal.pone.0303477 (PMC11135684; doi:10.1371/journal.pone.0303477)

**Evaluation of rapid diagnostic test kits for detection of *Treponema pallidum* antibody**

Sirinart Chomean^1,2^, Palakorn Puttaruk^3^, Phakawat Khamsophar^3^, Waraphorn Fukpo^3^, Chollanot Kaset^1,2*^

^1^Department of Medical Technology, Faculty of Allied Health Sciences, Thammasat University, Pathum Thani, Thailand

^2^Thammasat University Research Unit in Medical Technology and Precision Medicine Innovation, Thammasat University, Pathum Thani, Thailand

^3^Medical Technology Laboratory, Thammasat University Hospital, Thammasat University, Pathum Thani, Thailand

*Corresponding author

E-mail: chollanotk@gmail.com

S1 Fig. Discrepancies in the detection of syphilis antibodies among three distinct samples when comparing two prototypes of The Onsite Syphilis Ab Combo Rapid Test (prototypes Fd and Ff) with results from CMIA, RPR, TPPA, and the Determine™ Syphilis TP kit


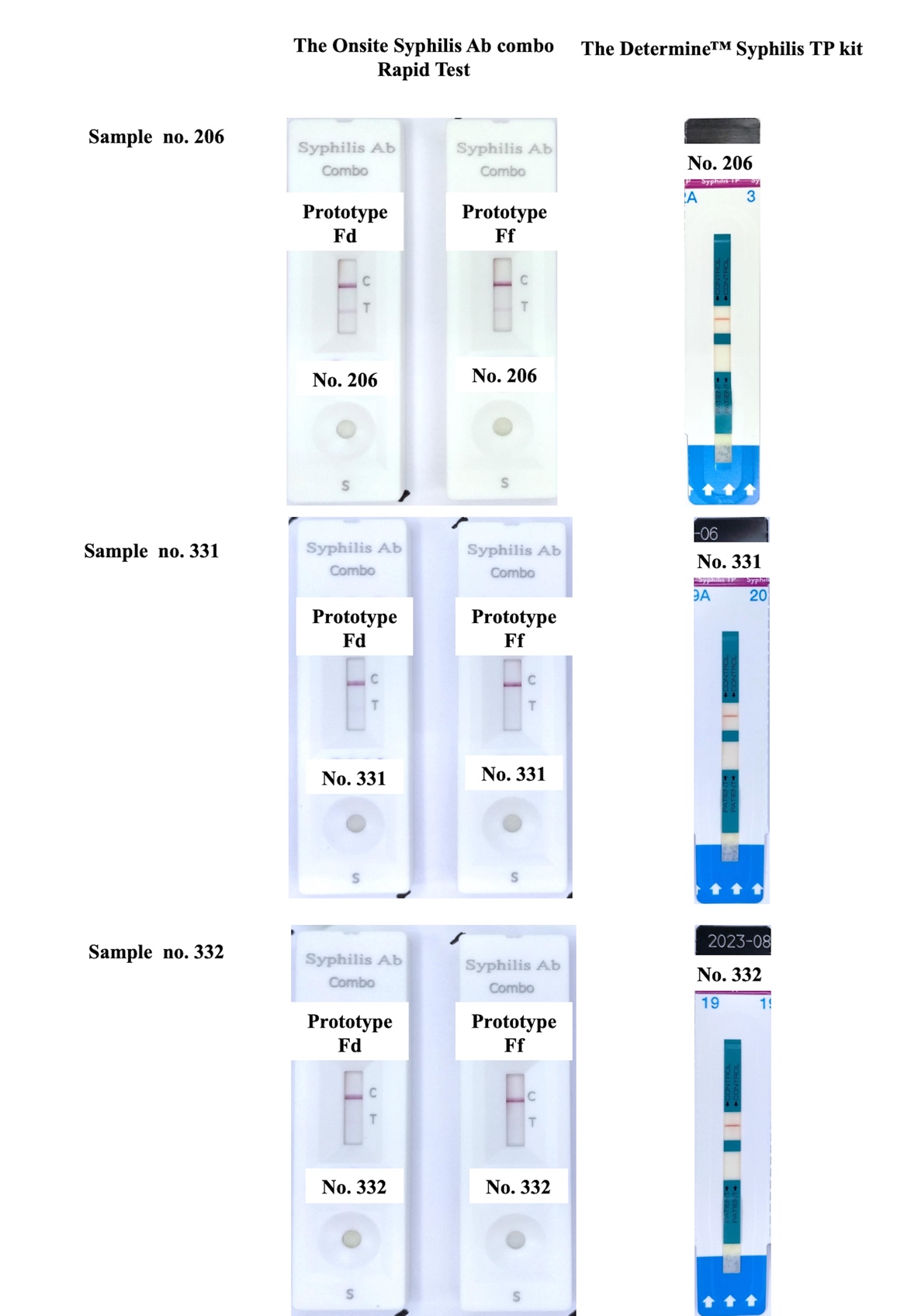

Supplement: S1 Fig — (DOCX) [file pone.0303477.s001.docx]
