# Supplementary material for: Evaluation of rapid diagnostic test kits for detection of Treponema pallidum antibody
Source: PLoS One. 2024 May 29;19(5):e0303477. doi: 10.1371/journal.pone.0303477 (PMC11135684; doi:10.1371/journal.pone.0303477)
Supplement: Attachment — Submitted filename: Reviewer Comments for authors attention.docx [file pone.0303477.s002.docx]

**Reviewer Comments to the authors**

Dear Authors,

Thank you for your study aiming to assess the accuracy and reliability of two new versions of the The Onsite Syphilis Ab Combo Rapid Test that demonstrated high sensitivity and specificity comparable to CMIA methods.

Please, consider the suggestions below to improve on this good study.

**Materials and Methods**

**Page 5, line 108.**

“Population Study”.

I think Study population or Study participants would be a much-preferred Subheading here.

**Page 6, line 113 -115**

“A total of 450 serum samples were collected from Thammasat University Hospital (TUH). These were classified into three groups: 200 reactive samples for T. pallidum antibodies obtained from syphilis patients…”

Please, how and with what were these samples classified? How was the diagnosis of ‘syphilis’ made in your study participants before samples were collected from them?

Please, give more details on your data collection procedures and sample collection and processing.

**Methods**

**Page 6, line 124-125**

“Each test utilized 40 µL of serum followed by 40 µL of the provided buffer.” If after 15-minute incubation at room temperature, results were visually interpreted, it then shows that that the buffer contains the recombinant antigen of the TP bacterium. If this is the case, please indicate that the buffer contains the antigen, or that both buffer and the recombinant antigen were added also.

**Correlation analysis of** **The Onsite Syphilis Ab combo Rapid Test with CMIA, RPR and TPPA test**

**Page 8, line 170-171**

“Onsite Syphilis Ab Combo Rapid Test were grouped into W+, 2+, and 4+”. Please explain what W+ stands for and rationale behind the grouping into +W, 2+, and 4+.

**Results**

**Page 9, line 196**

“…as illustrated in Table 1”.

Please, where is the Table 1 mentioned here?

**Also, consider preparing a three-column table displaying overall sensitivity, specificity, predictive values, and likelihood ratios of The Onsite Syphilis Ab combo Rapid Test using CMIA as the standard**.

| **Measures of Diagnostic accuracy (Or Diagnostic Parameters)** | **Estimated values (%)** | **95% Confidence intervals** |
| --- | --- | --- |
| Sensitivity |  |  |
| Specificity |  |  |
| PPV |  |  |
| NPV |  |  |
| PLR |  |  |
| NLR |  |  |
| Accuracy |  |  |

**Page 12, line 244**

“…illustrated that its performs effectively across various…”

Please, correct to “…illustrated that it performs effectively across various…”

**Page 15, line 272-276**

“In conclusion, the Onsite Syphilis Ab combo Rapid Test (prototype Ff) demonstrates a

high degree of efficacy in detecting syphilis infections…”

I do not understand why your conclusion tend to project the performance of Prototype Ff over Fd while in your analysis, Prototype Fd out-performed Prototype Ff. I would suggest you reverse this conclusion in favour of Prototype Fd. Prototype Fd consistently gave strong positive reactions (100%) when compared with Ff (87.5%) against different TPPA tires.

Please, what do you think are the major limitations of this study? Please, add this before your conclusion.

**Reference**

**Page 24, line 465, reference no 15**

“…Wilson C, Peterman TA. 2019. Frequency and characteristics of biological false-positive test results…”

Please remove 2019 if it’s not part of the journal article title.
